# A Prospective Observational Study of Endotracheal Intubation Practices in an Academic Emergency Department of a Tertiary Care Hospital in South India

**DOI:** 10.7759/cureus.36072

**Published:** 2023-03-13

**Authors:** Kumaresh P Tamilarasu, Arshiya Aazmi, Stalin Vinayagam, Gunaseelan Rajendran, Sanket Patel, Bahiya Aazmi

**Affiliations:** 1 Department of Emergency Medicine, Karpaga Vinayaga Institute of Medical Sciences, Chengalpattu, IND; 2 Department of Emergency Medicine, SDM College of Medical Sciences and Hospital, Dharwad, IND; 3 Department of Anaesthesiology, Jawaharlal Institute of Post Graduate Medical Education and Research (JIPMER), Puducherry, IND; 4 Department of Emergency Medicine, Aarupadai Veedu Medical College & Hospital, Puducherry, IND; 5 Department of Emergency Medicine, Nootan Medical College and Research Centre, Gujarat, IND; 6 Department of Neurorehabilitation, National Institute of Mental Health and Neurosciences, Bengaluru, IND

**Keywords:** first-pass success, emergency endotracheal intubation, indian emergency department, advanced airway management, rapid sequence intubation

## Abstract

Introduction: Airway management is the first critical step to be addressed in the airway, breathing, and circulation algorithm for stabilizing critically ill patients. Since the emergency department (ED) is the primary contact of these patients in health care, doctors in the ED should be trained to perform advanced airway management. In India, emergency medicine has been recognized as a new specialty by the Medical Council of India (now the National Medical Commission) since 2009. Data related to airway management in the ED in India is sparse.

Methods: We conducted a one-year prospective observational study to establish descriptive data regarding endotracheal intubations performed in our ED. Descriptive data related to intubation was collected using a standardized proforma that was filled by the physician performing intubation.

Results: A total of 780 patients were included, of which 58.8% were intubated in the first attempt. The majority (60.4%) of the intubations were performed in non-trauma patients and the remaining 39.6% in trauma patients. Oxygenation failure was the most common indication (40%) for intubation followed by a low Glasgow coma scale (GCS) score (35%). Rapid sequence intubation (RSI) was performed in 36.9% of patients, and intubation was done with sedation only in 36.9% of patients. Midazolam was the most commonly used drug - either alone or in combination with other drugs. We found a strong association of first-pass success (FPS) with the method of intubation, Cormack-Lehane grading, predicted difficulty in intubation, and experience of the physician performing the first attempt of intubation (P<0.05). Hypoxemia (34.6%) and airway trauma (15.6%) were the most commonly encountered complications.

Conclusion: Our study showed an FPS of 58.8%. Complications were seen in 49% of intubations. Our study highlights the areas for quality improvement in intubation practices in our ED, like the use of videolaryngoscopy, RSI, airway adjuncts like stylet and bougie, and intubation by more experienced physicians in patients with anticipated difficult intubation.

## Introduction

The emergency department (ED) functions as a primary contact for critically ill and severely injured patients in tertiary care hospitals and teaching institutions. These critically ill patients are managed by both emergency medicine physicians (EMPs) and doctors of various specialties. The recent development of emergency medicine (EM) as a recognized branch of medical specialty has resulted in many teaching institutions having EM residency programs. ED is well established in developed countries like Australia, UK, and USA [1-5]. But in India, it is in a developing stage after the recognition of the specialty by the Medical Council of India (now National Medical Commission) in 2009 [6].

Patients with hemodynamic instability will collapse unless resuscitated immediately. They can be safely stabilized by airway, breathing, and circulation algorithm. The airway is the first critical step to be addressed and one of the cornerstones of effective resuscitation of critically ill and severely injured patients [1]. These patients are managed with advanced airway techniques like endotracheal intubation or surgical airway. Intubations done in ED are often challenging due to multifactorial reasons like facial deformity, trauma, physiological instability, and the need for cervical spine immobilization in suspected spine trauma patients. The incidence of difficult intubations is high in ED (3.0%-5.3%) when compared to operating rooms (1.15%-3.8%) [4]. In developing countries, airway management is mostly done by anesthesiologists. Data about emergency airway management is sparse in India. We conducted this prospective observational study to describe the ED intubation practices in a level-one trauma center in South India with respect to factors associated with the success of endotracheal intubation, medications used, and complications of intubation.

This article was previously presented as an oral presentation of original research at the 2019 AETLS National Level Conference on March 23, 2019.

## Materials and methods

After obtaining institutional ethics committee approval, this prospective observational study was conducted in a tertiary care teaching hospital in South India for a period of one year, from July 2017 to June 2018. More than 92,000 patients visited our ED for treatment during our study period, and 6% of patients belonged to Emergency Severity Index level one and level two categories requiring resuscitation and emergent care. Our ED has a three-year MD EM training program. During the training period, residents in the first, second, and third years of training (PGY1, PGY2, and PGY3, respectively) work under qualified EMPs with three or more years of experience in the ED. ED intubations were performed by EM residents and residents of other clinical departments posted in the ED as part of their peripheral postings, under guidance and monitoring by the attending EMP on duty. Written informed consent was sought from relatives of those patients who underwent intubation in our ED for the collection of relevant data for the purpose of our study. The patients whose relatives consented were included. The study excluded patients aged <18 years, patients who were intubated outside and referred to our hospital for further management, pregnant patients, and those who did not consent to participation in the study. Descriptive data related to intubation was collected using a standardized proforma that was filled by the physician performing intubation. This was done at the time of documentation of procedure notes following the procedure to minimize recall bias. LEMON criteria were used to predict difficult intubations, and Cormack-Lehane grading was performed during direct laryngoscopy.

The following data were collected:

Patient demographics

Age and gender are the collected patient demographics of this study.

Patient clinical characteristics

Vital Signs of the Patient

The collected vital signs of the patient include pulse rate, blood pressure, oxygen saturation, and respiratory rate.

Primary/ Provisional Diagnosis of the Patient at the Time of Intubation

Assessment of LEMON criteria: It was done whenever possible, depending on the clinical condition of the patient.

L - Look externally (facial trauma, large tongue, large incisors, beard, or mustache)

E - Evaluate the 3-3-2 rule

M - Mallampati score>3

O - Obstruction or obesity

N - Restricted neck mobility

Intubation characteristics

Indication of Intubation

This included one or more of the following: loss of airway patency - inability to handle his or her secretions and inability to phonate in response to voice command, failure to oxygenate or ventilate - persistent hypoxemia despite maximal oxygen supplementation (SpO_2_<90%) or ventilatory failure that is not reversible by clinical means, low Glasgow coma scale score (GCS<8), and elective indications of intubation - anticipated airway deterioration or need for intubation to facilitate a patient’s evaluation and treatment.

Method of Intubation

Rapid sequence intubation (RSI): Simultaneous administration of an induction agent and a neuromuscular blocking agent to facilitate tracheal intubation.

Intubation with sedation: Use of sedative agents like ketamine, propofol, or benzodiazepines, without the use of a neuromuscular blocking agent.

Crash intubation: Intubation done in patients in cardiopulmonary arrest, deep coma, or near death, who can't maintain ventilation and oxygenation without the assistance of induction and neuromuscular blocking agents.

Awake intubation: Intubation in an awake, spontaneously breathing patient with an anticipated difficult airway, assisted by local anesthetic and/or conscious sedation.

Medications Used

Sedatives: Midazolam (0.1-0.2 mg/kg), propofol (0.5-1.5 mg/kg), ketamine (1-2 mg/kg), and fentanyl (3 µg/kg).

Neuromuscular blocking agents: Succinylcholine (1.5 mg/kg).

Number of Attempts at Intubation

An "attempt" is defined as a single advanced airway maneuver beginning with the insertion of the laryngoscope into the patient’s mouth and ending when the device is removed.

First-pass success (FPS) is defined as successful endotracheal intubation at the first attempt of direct laryngoscopy.

Designation or Training Level of the Physician Performing the Intubation

ED intubations were performed by EM residents and residents of other clinical departments posted in the ED as part of their peripheral postings, under guidance and monitoring by the attending EMP covering the shift.

Designation or training level of the physician performing the intubation includes EM residents in PGY1, PGY2, and PGY3; attending EMP with three or more years of experience in ED; and others include residents of other clinical departments posted in the ED as part of their peripheral postings.

Complications Related to the Procedure

Hypoxemia (defined as spO_2_<90%), hypotension (defined as systolic BP<90 mmHg), esophageal intubation, cardiac arrest, vomiting with or without aspiration, and airway trauma/dental trauma due to intubation are the complications related to the procedure.

Data analysis

Statistical analysis was performed using SPSS version 19 (IBM SPSS Statistics for Windows, Version 19.0. Armonk, NY: IBM Corp). Continuous variables were expressed as mean with standard deviation or median with range, and categorical variables were expressed as frequency with percentage. The association between categorical variables was analyzed by the chi-square test or Fischer exact test. The association of continuous variables with outcome was tested using either the parametric t-test, Pearson correlation, or non-parametric tests like Spearman rank correlation, depending upon the outcome distribution. Statistical analysis was carried out with a 5% level of significance. P value <0.05 was considered significant.

## Results

Descriptive data from 780 ED intubations was collected during our study period. Of 780 intubations, the majority (60.4%) of the intubations were performed in non-trauma patients and the remaining 39.6% in trauma patients. The mean age of study participants was 46.74 years, and the mean weight was 66.93 kg. 

**Figure 1:**
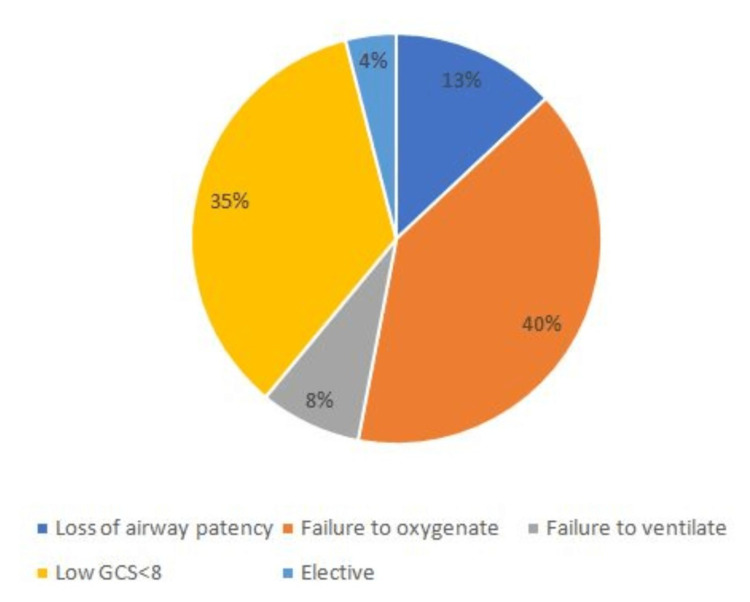
Distribution of the Study Population Based on Indication of Intubation GCS, Glasgow coma scale

The most common indication for intubation was the failure to oxygenate (40%) followed by low GCS<8 (35%), as seen in Figure 1.

The most common methods of intubation used were RSI and intubation with sedation, as mentioned in Table 1. When intubation was performed with sedation alone, 92 (36%) intubations were performed with midazolam+fentanyl, and 65 (25%) intubations were performed with ketamine. Succinylcholine was the paralytic agent used in this study. Midazolam+fentanyl was used in 114 (39.6%) patients who underwent RSI, and propofol+fentanyl was used in 56 (19.4%) patients. The mean duration taken to intubate the patients using RSI was lower (3.45+1.59 minutes) as compared to the patients intubated with sedation alone (4.35+1.95 minutes).

**Table 1:** Distribution of the Study Population Based on the Method of Intubation

Method of intubation	Number of patients (n=780)	Percentage (%)
Rapid sequence intubation	288	36.9
Intubation with sedation	288	36.9
Crash intubation	131	16.8
Awake intubation	73	9.4

**Figure 2:**
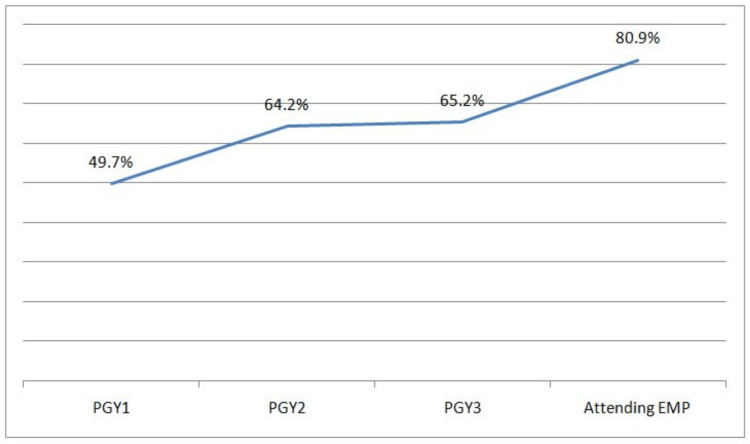
First-Pass Success Based on Experience Level of the Physician Performing Intubation PGY1, emergency medicine residents in the first year of postgraduate training; PGY2, emergency medicine residents in the second year of postgraduate training; PGY3, emergency medicine residents in the third year of postgraduate training; EMP, emergency medicine physician

Stylet was used in 133 (17.1%) intubations and bougie in 121 (15.5%) intubations. Based on LEMON criteria, 207 patients (26.5%) were predicted to have a difficult airway. FPS was seen in 459 (58.8%) intubations, and 705 (90.3%) intubations were successful within two attempts. The FPS increased with the experience level of the physician performing intubation, as seen in Figure 2. Predicted difficult airway, Cormack-Lehane grade 1 and grade 2, RSI, and higher experience level of the intubator were significantly associated with FPS (P<0.05).

Of the total 780 patients, 382 (49%) patients experienced at least one complication. Hypoxemia was the most common complication occurring in 270 (34.6%) patients followed by airway trauma in 122 patients (15.6%). Hypotension occurred in 41 (5.2%) patients, and it was most common in patients intubated with propofol.

## Discussion

The FPS of ED intubations in our study was 58.8%. This is lower than the cut-off of 84.1% reported by a systematic review and meta-analysis of 16 publications from 10 countries, which serves as a benchmark for emergency airway performance [7]. This is also lower than the FPS reported by a study conducted by Jadhav P et al. in an Indian ED (77.19%), which is the only available study reported on ED intubation practices in India [1]. Recent multicenter studies from Ireland and China have reported an FPS of 89% and 85.7%, respectively [8,9].

Of the 780 intubations, 49% were associated with complications. This is high compared to that reported by various international studies ranging from 4.2% to 26% [10-12]. Jadhav et al. reported that 33.33% of patients experienced complications [1].

The lower FPS and higher complications in our study could be attributed to various factors, including less experience of physicians performing intubation since the majority of intubations in our study were performed by EM residents. We used direct laryngoscopy for all intubations due to the non-availability of a videolaryngoscope in our ED. RSI was used in a lesser proportion of patients as compared to other studies. Airway adjuncts like stylet and bougie were used in a lesser proportion of patients as compared to other studies.

The majority (91.92%) of ED intubations in our study were done by EM residents. This is different from other studies, which had the majority of intubations performed by more experienced physicians like attending EMPs or anesthesiologists or intensive care medicine doctors. A recent multicenter study of 41 EDs from China reported complications in 19% with an FPS of 89% [9]. In this study, 35.4% of ED intubations were performed by residents. Another recent study from Columbia reported that EM residents only performed 1.3% of intubations, and the remaining were performed by more experienced physicians. This study reported complications in 9% and an FPS of 90.5% [13]. A multicenter observational study conducted across 11 Irish EDs reported that 90% of intubations were performed by physicians at the registrar level. This study reported complications in 19% and 89% of FPS [8].

All the intubations in our study were performed using direct laryngoscopy due to the non-availability of a videolaryngoscope. The use of videolaryngoscopy was reported in 53% of intubations in an Irish multicenter study with an FPS of 89% [8]. The FPS was significantly higher with the use of videolaryngoscopy (78%) when compared to direct laryngoscopy (58%), as reported by a Japanese study of 287 emergency intubations [14]. Videolaryngoscope is proven to have a better outcome and higher FPS rate compared to direct laryngoscope in a systematic review by Lewis et al. [15]. This is particularly helpful in obese patients in whom there is difficulty in patient positioning and aligning the oral, pharyngeal, and laryngeal axes.

RSI was performed only in 36.9% of intubations, which is lower when compared to other studies. A multicenter observational study in 13 Japanese EDs analyzed the effectiveness of RSI versus non-RSI in the ED and reported statistically significant FPS with RSI compared to non-RSI (73% v/s 63%) [16].

Our study reported the use of airway adjunct devices like stylet and bougie in 17.1% and 15.5% of intubations, respectively. A randomized clinical trial on 757 intubations reported that bougie use resulted in significantly higher FPS than a stylet (96% vs. 82%) for patients undergoing ED intubations [17]. Though 92.98% of ED intubations were performed by EM residents in the Indian study by Jadhav P et al., all the intubations in their study were non-RSI, and they had a higher FPS (77.19%). This could be attributed to the use of bougie in 61.4% of intubations, as opposed to 15.5% in our study [1].

In our study, those predicted with a difficult airway had statistically significant lower FPS (41.1%) compared with those predicted to have no difficulty (65.4%). Hence, predicted difficult intubations will have a better FPS if performed by more experienced physicians; with the use of rapid sequence intubation; and with the use equipment like bougie, stylet, and videolaryngoscope.

## Conclusions

Our study showed an FPS of 58.8%. Complications were seen in 49% of intubations. The majority of intubations were performed by lesser experienced emergency care providers (EM residents). The use of RSI and equipment like stylet and bougie, which is considered standard practice in western countries, was seen only in 17.1% and 15.5%, respectively. Our study highlights the areas for quality improvement in intubation practices in our ED like the use of videolaryngoscopy, RSI, airway adjuncts like stylet and bougie, intubation training (supervised intubations, simulation, and classroom training), and intubation by more experienced physicians in patients with anticipated difficult intubation. The lack of availability of sufficient data from Indian EDs necessitates the maintenance of national airway registries to understand ED intubation practices in India.
